# Skeletal Muscle as an Auto-, Para- and Endocrine Organ: The Role of Myokines in Muscle Metabolism and Other Metabolic Organs

**DOI:** 10.33549/physiolres.935751

**Published:** 2025-12-01

**Authors:** Luděk HORVÁTH, Matej PEKAŘ, Zdeněk ŠVAGERA, Veronika HORKÁ, Miloš MRÁZ, Marek BUŽGA

**Affiliations:** 1 Diabetes Centre, Institute for Clinical and Experimental Medicine, Prague, Czech Republic; 2 Department of Physiology and Pathophysiology, Faculty of Medicine, University of Ostrava, Ostrava, Czech Republic; 3 Department of Physiology, Faculty of Medicine, Masaryk University, Brno, Czech Republic; 4 Vascular Surgery, Hospital AGEL Trinec-Podlesi, Trinec, Czech Republic; 5 Institute of Laboratory Medicine, University Hospital Ostrava, Ostrava, Czech Republic; 6 Department of Human Movement Studies, Pedagogic Faculty, University of Ostrava, Ostrava, Czech Republic; 7 Department of Medical Biochemistry and Laboratory Diagnostics, First Faculty of Medicine, Charles University, Prague, Czech Republic

**Keywords:** Skeletal muscle cells, Myokines, Secretome, Autocrine effect, Paracrine effect

## Abstract

Skeletal musculature represents the largest organ in the human body, playing a vital role in systemic metabolism, physiological functions, and glucose homeostasis. Skeletal muscles are also a significant source of multiple humoral factors, including myokines, which are, as part of the muscular secretome, involved in cellular signaling within and outside of the muscle. Myokines are a group of cytokines that exert a major influence on muscle metabolism through autocrine mechanisms and are involved in para- or endocrine regulation in organs outside of muscle tissue, such as the pancreas, adipose tissue, liver, heart, bone, gastrointestinal tract, and brain. In the future, these findings could be crucial for the identification of important biomarkers used for the monitoring of physical activity in the treatment of pathologies such as intensive care-associated muscle wasting, sarcopenia, diabetes, neurodegenerative diseases, etc. As skeletal muscle tissue is intrinsically linked to multiple types of tissues and organs metabolically, functionally, and most importantly, regionally, there can be a significant overlap between the auto- and paracrine effects of myokines, depending on the presence of that myokine. The following section will discuss the auto-, para-, and endocrine effects of some of the myo-inducible cytokines on skeletal muscles and adjacent tissue types.

## Introduction

The skeletal musculature of the human body can be considered to be the largest and heaviest organ of the human body. It makes up approximately 40 % of the total body weight and contains the largest protein deposit in the body, accounting for 50–75 % of all body proteins [1]. This complex organ has a wide spectrum of functions that include participation in immune-metabolic processes by producing molecules that are important for the immune system, while also functioning as a buffer for glucose utilization. However, detailed descriptions of most of these mechanisms have eluded us for many decades, and skeletal muscle has been primarily viewed as a chemical-to-mechanical energy-transfer system. The first impulse in exploring the role of skeletal muscle in endocrine regulation came in 1961, originating from Goldstein’s group’s *in vivo* experiment on canine models involving metabolic experiments on skeletal muscle both at rest and after stimulation by electrical activity. These canine experiments have shown that the glucose utilization rate was higher than could be expected just from local muscular mechanical activity alone and hinted towards a more complex humoral mechanism at work [2].

The first molecule that was described in 1983 and associated with skeletal muscle was a molecule that the authors at the time believed had a pyrogenic effect [3]. Interestingly, the molecule in question was identified to be present after cycling exercise, but detailed histological analysis of its expression in post-exercise skeletal muscle was not performed until 1998 by Ostrowski’s group [4]. The conceptualization of skeletal muscle as an endocrine organ capable of secretion of specific molecules, termed “myokines”, was introduced in 2003 by Pedersen’s group [5].

The name myokine is composed of two Greek words: *myo*, muscle, and *kine*, agonist. Many of the myokines described are formed during muscular work and are important in the acute response to muscle loading and in the adaptation to chronic loading [6]. Myokines have a major influence on muscle metabolism through autocrine mechanisms and para- or endocrine regulation in organs outside of muscle tissue, such as the pancreas, adipose tissue, liver, heart, bone, gastrointestinal tract, and brain [7].

Studies in recent years have shown that myokine signaling can be attenuated in response to external and internal stimuli, that include dyskinesia, ageing, and even certain specific types of diets, including high-fat diets. These changes are likely related to disease exacerbations and age-related changes to bodily functions and thus are important in the search for biomarkers usable in the prevention of frailty and sarcopenia syndrome. Therefore, myokines may play a critical role in disrupting pathological mechanisms related to ageing and related disorders such as muscle atrophy, sarcopenia, diabetes, and chronic inflammation in adipose and muscle tissue [8].

## Skeletal muscle as a secretory organ

**Fig. 1:**
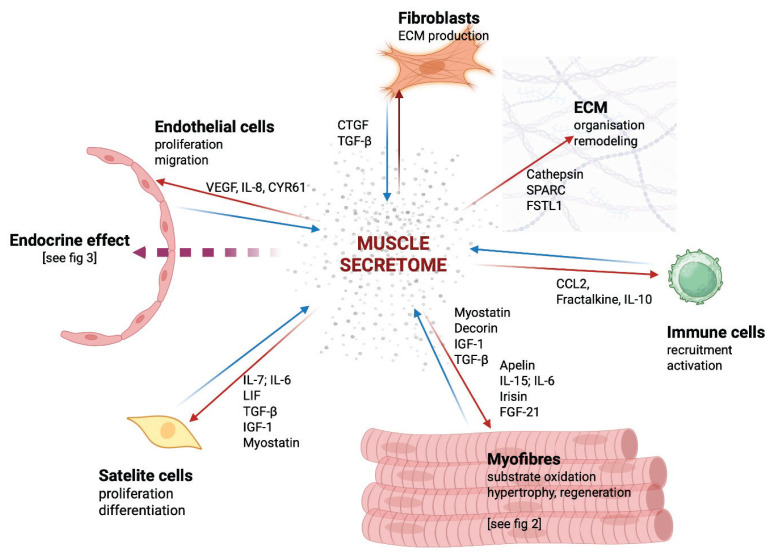
Muscle secretome and para and autocrine effects of human myokines. CCL2 – C-C motif chemokine ligand 2, CTGF – connective tissue growth factor, CYR61 – cysteine-rich angiogenic inducer 61, ECM – extracellular matrix, FGF-21 – fibroblast growth factor 21, FSTL-1 – follistatin-like 1, IGF-1 – insulin-like growth factor 1, IL-10 – interleukin-10, IL-15 – interleukin-15, IL-6 – interleukin-6, IL-7 – interleukin-7, IL-8 – interleukin-8, LIF – leukemia inhibitory factor, SPARC – secreted protein acidic and rich in cysteine, TGF-β – transforming growth factor beta, VEGF – vascular endothelial growth factor.

Skeletal muscle produces a large number of molecules, a collection of which is called the muscle secretome. While up to 3000 molecules have been described as potential myokines [9], recent studies utilizing muscle secretome-targeted proteomic analyses have identified 654 specific proteins and peptides that can be identified as myokines [10]. At the moment, only a select group of myokines, such as IL-6, Irisin and Myostatin, have their endocrine function in the human body described in more detail [11]. In addition to peptides and proteins, the secretome also includes molecules such as lactic acid and amino acids [12], microRNAs and nucleic acids, including mitochondrial DNA [13]. Interestingly, some proteins are not formed in a potentially secretory form but are transported in microvesicles, such as exosomes [14]. An overview of the basic relationships and selected molecules of the muscle secretome is shown in Figure 1.

## Auto and paracrine myokine function in skeletal muscle

**Fig. 2:**
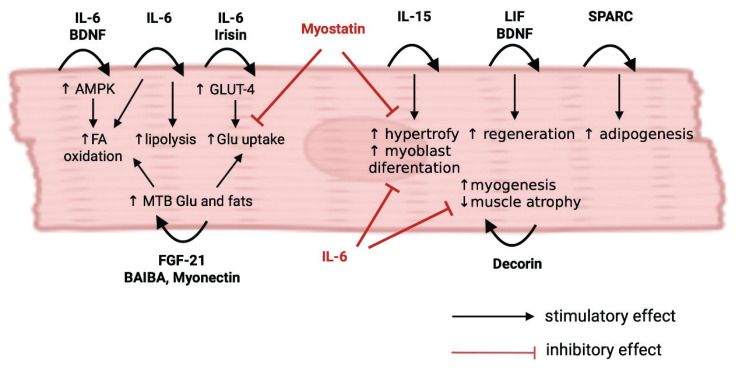
Autocrine effects of myokines in skeletal muscle. AMPK – AMP-activated protein kinase, BDNF – brain-derived neurotrophic factor, BAIBA – β-aminoisobutyric acid, FA – fatty acids, FGF-21 – fibroblast growth factor 21, GLUT-4 – glucose transporter type 4, IL-6 – interleukin-6, IL-15 – interleukin-15, LIF – leukemia inhibitory factor, MTB – mitochondrial beta-oxidation, SPARC – secreted protein acidic and rich in cysteine.

As the importance of autocrine, paracrine, and endocrine functions of skeletal muscle is brought to the fore, it becomes apparent that skeletal muscle is, in a similar way to other major endocrine organs, subject to its own complex humoral regulation and feedback loops. In contrast to other endocrine tissues, the precise mechanisms of myokine action in skeletal muscle have not been sufficiently specified so far. This is in part due to the methodological heterogeneity of the studies, the unclear (or multiple) origin sites during physical activity, and the pleiotropic action of the studied cytokines [6]. As skeletal muscle is intrinsically linked to multiple types of tissues and organs metabolically, functionally, and, more importantly, regionally, there can be a significant overlap between autocrine, paracrine, and endocrine effects of myokines, depending on the presence of that myokine in the body. This situation is made more complex by the fact that some myokines are released only into the systemic circulation at a high enough concentration during vigorous enough exercise, providing no or only limited paracrine effect during rest. As skeletal muscle tissue is surrounded by a multitude of other types of tissues and organs, including peripheral nerves, adipose tissue, and other connective and fibrous tissues (specifically bones, tendons, and ligaments), the following section will discuss autocrine, paracrine, and endocrine effects of myokines (and, in some cases, exerkines – e.g. substances released during exercise) on skeletal muscle and these neighboring tissue types. A summary and description of the myokines listed can be found in Figure 2 and Table 1.

## Interleukin-6 (IL-6)

Although IL-6 is primarily associated with its pro-inflammatory function, it was also the first myokine to be named as such in the literature [5]. The pleiotropic and sometimes contradictory effects of IL-6 on human physiology are derived primarily from differences in three main IL-6 pathways: cis (or classical), trans, and cluster signalization [15]. Although the typically anti-inflammatory cis signalization is transduced through the membrane IL-6 receptor and leads to subsequent activation of gp130, trans signalization is mediated by the interaction between IL-6 and soluble IL-6 receptors (sIL-6R) in the extracellular matrix, which then induces a pro-inflammatory response through a membrane-bound gp130, both of which lead to phosphorylation of the signal transducer and activator of transcription 3 (STAT3) [16]. Cluster signalization is spatially more complex, involving an interaction between two cells and the IL-6 molecule. IL-6 cis signaling is present only in selected organs, including skeletal muscle and the liver, while trans signaling is present more ubiquitously and is associated with a pathological response [15]. Although skeletal muscle has constant expression of the IL-6 receptor, physical exercise leads to its significant increase, which appears to contribute to the specificities of IL-6 action in response to physical activity [17].

Although it was originally thought that the increase in IL-6 concentration in the bloodstream after physical exercise was an inflammatory response to muscle damage and/or metabolic stress, immunohistochemistry staining has shown that IL-6 is expressed by both skeletal muscle myocytes and interstitial cells [18].

Although the endocrine effect of IL-6 has been well described in part due to its role in the therapy of autoimmune and rheumatic diseases, the paracrine effect of IL-6 after exercise was studied less extensively. Paracrine secretion of IL-6 by growing myotubes has been theorized to be necessary for skeletal muscle hypertrophy in mice [19]. However, in seeming contradiction to *in vitro* experiments, acute systemic application of high-dose IL-6 leads to muscle breakdown in rats [20], with long-term infusion leading to muscular atrophy [21].

The significantly different effects of IL-6 on skeletal muscle are further complicated when taking into account the effect of IL-6 on human metabolic activity, as systemic infusion of IL-6 in a concentration similar to those found after vigorous exercise leads to a reduction in arterial amino acid concentration despite increased breakdown of skeletal muscle [22]. These ramifications for energy metabolism are further expanded by the effect of exercise-induced IL-6 on human adipose tissue, leading to mobilization of free fatty acids [23] and reduction in visceral adipose tissue [24], which can be antagonized by the application of the IL-6 antibody.

Furthermore, IL-6 also appears to have a potential paracrine function in the energetic shift and energy modulation of skeletal muscle during exercise, as *in vitro* addition of IL-6 to murine myotubes has been found to increase glucose uptake and lead to oxidation of fatty acids in skeletal muscle through the AMPK pathway, further cementing the effect of muscle-derived IL-6 on energy metabolism after physical exercise [25].

Taken together, it could be postulated that while paracrine IL-6 signaling is necessary for a physical activity-dependent energy change and leads to muscle hypertrophy in murine myotubes [25], IL-6 endocrine activity has a role to play in human systemic energy and amino acid metabolism [26], which could be the main cause of the apparent disparity in the effect of IL-6 between *in vivo* and *in vitro* studies.

Since most studies on IL-6 focus on prolonged exposures or acute high doses of IL-6 and therefore predominantly induce trans signaling, it is difficult to determine what the paracrine effect would be in surrounding tissues, and more studies are needed on the local effects of muscle-derived IL-6.

## Brain-derived neurotrophic factor (BDNF)

BDNF is a cytokine belonging to the neurotrophin family, produced primarily by the central nervous system, though a significant fraction is produced by the skeletal muscle [27]. The action of BDNF is transduced through the tropomyosin receptor kinase B receptor (TrkB), which has been detected both in neurons and skeletal muscle cells [27]. BDNF is released by skeletal muscle in response to exercise, although muscle-derived BDNF does not appear to be released into the circulation at a high concentration, as increased concentration of BDNF after exercise has been observed mainly in the muscle itself [28]. However, data are inconclusive in this regard, and some studies have demonstrated a significant increase in BDNF after physical exercise, even in systemic circulation [29]. Although the presence of BDNF in the central nervous system is necessary for neurogenesis and synaptic connectivity [30], its effect on skeletal muscle is exercised both at the neuromuscular junction [31] and skeletal muscle itself [32].

In skeletal muscle, BDNF appears to be necessary for the regulation of energy metabolism of skeletal muscle, as BDNF deficiency at the cell level leads to reduced fatty acid-induced mitofission and to mitophagy [33]. At the macroscopic level, this translates to body weight gain, myosteatosis, and worse metabolic flexibility in examined mice, which can be rescued by the use of a BDNF mimetic [33]. Furthermore, BDNF has been shown to be highly effective in mimicking the effect of physical exercise on the skeletal muscle [34], underlining its role in muscular physiology and showing potential therapeutic benefits.

From a speculative point of view, BDNF also appears to have a positive effect on bone density, as *in vivo* experiments have shown that BDNF stimulates osteoblasts through its TrkB receptor and a TrkB agonist has been shown to have a therapeutic effect in osteoporosis [35]. However, since the systemic release of BDNF has not been concretely proven and the paracrine effect of muscle-derived BDNF on bone has not been studied, the presence of a physiological muscle-bone crosstalk through BDNF is debatable. BDNF also appears to play a role in the regulation of adipose tissue, as heterozygous mice (BDNF^+/−^) have been found to have different adipokine profiles and altered plasma levels of BDNF. However, since the primary metabolic action of BDNF lies in the CNS, which would also be affected by chronic BDNF deficiency, the correlation between plasmatic BDNF values and its effect on adipose tissue is difficult to establish [36].

When taken together, the data from these trials show a highly complex and pleiotropic effect of BDNF on the autocrine or paracrine level not only in skeletal muscle itself, but also in facilitating neuromuscular connection [31]. These findings suggest that BDNF may play a crucial role in skeletal adaptation to physical activity, seemingly through paracrine mechanisms, as the data on the systemic release of muscle-derived BDNF is still inconclusive.

## Irisin

Irisin, a cleaved product of membrane-bound FNDC5 (fibronectin domain III containing 5), is a myokine with autocrine, paracrine, and endocrine effects. Although the receptor in skeletal muscle has not yet been confirmed, it has been shown that irisin exerts its action in bone, hippocampus, and adipose tissue through αV integrin receptors V, specifically αVβ5 [37]. As inhibition of αVβ5 led to an attenuated effect of irisin in skeletal muscle, the integrin αVβ5 also appears to be an integral part of irisin signaling in skeletal muscle. Irisin is produced by skeletal muscle in the resting state, with a significant transient increase after physical activity [38].

*In vitro* activation of skeletal muscle by irisin leads to a significant proliferation of murine myoblasts and a higher concentration of activated myoblast nuclei in a dose-dependent manner, while also accelerating muscle regeneration and attenuating muscle atrophy after denervation in mice [39]. Injection of irisin in mice also leads to significant macroscopic and functional changes, such as gain in body weight and increased grip strength [39]. When taken together, these findings show a significant paracrine effect of muscle-derived irisin on skeletal muscle.

Irisin has also been attributed with a beneficial effect on metabolism, as high doses of irisin (3,500 μg/kg/week) have been associated with the browning of adipose tissue in mice [40]. While available data in humans shows a significant effect of irisin on long-term energy metabolism and exercise capacity [41], the current evidence does not show an acute effect of exercise-induced irisin on energy metabolism.

Regarding the effect of irisin on connective tissue, even low doses of irisin (100 μg/kg per week) have led to bone strengthening in mice, specifically in terms of mineral density of the cortical tissue, periosteal circumference, polar moment of inertia, and bending strength [40]. Irisin has also been found to have a positive effect on tendons *in vitro*, showing a proliferative and anti-inflammatory effect on hamstring tendons harvested from human patients undergoing anterior cruciate ligament reconstruction [42].

Irisin may also play a role in the neuroprotective effect of physical exercise, as the application of irisin is associated with a reduction in apoptosis in the CNS and an increase in BDNF concentration in the rodent cortex [43]. As BDNF also has a local paracrine effect on the neuromuscular junction [31], it remains to be seen whether the increase in irisin-induced BDNF production is specific to the cortex or whether irisin also increases the local concentration of BDNF in the periphery.

Although the effect of irisin on the peripheral nervous system has not been adequately explored, a one-time injection of irisin has acutely decreased the thermal threshold in diabetic mice after 60 min during the heat withdrawal test [44]. However, no conclusion can be drawn on the long-term effect of irisin on diabetic neuropathy from this trial.

## Myostatin

Myostatin’s apparent primary function in the human body is its role as the master muscle mass regulator, preventing excessive muscle hypertrophy through a negative feedback loop. While myostatin is expressed and secreted primarily by skeletal muscle tissue, it is also produced in smaller quantities by adipose tissue [45], cardiomyocytes [46], kidneys, and the central nervous system [47].

Myostatin is a member of the TGF-β family that exerts its action through activin receptor type IIB receptors, which after activation form a receptor complex with activin receptor-like kinase 4 (ALK4) or ALK5 (Type I receptors), leading to the phosphorylation of transcription factors Smad2 and Smad3 [48]. Phospho-rylated Smad 2/3 then stimulates FoxO-dependent transcription and induces muscle protein breakdown through the ubiquitin proteasome system and increased autophagy [49].

The effect of myostatin on skeletal muscle has been well demonstrated *in vitro*, since the addition of myostatin to murine myofiber explant cultures inhibits satellite cell activation [50] and leads to myotubular degeneration in human skeletal muscle cell culture [51]. When considered *in vivo*, myostatin in skeletal muscle leads to loss of skeletal muscle mass, as myostatin deficiency leads to significant skeletal muscle hypertrophy in myostatin-knockout animals compared to wild-type animals [52]. However, the knock-out of myostatin itself can have wildly differing consequences in various animals, as murine myostatin knockouts have been shown to increase muscle mass by up to 200–300 %, compared to a more modest 20–25 % increase in skeletal muscle mass in cattle [52]. Our knowledge concerning the benefits of myostatin inhibition is also close to the point of clinical benefit, as myostatin inhibition (either through ligand traps, overexpression of specific antagonists, such as follistatin, or *via* small molecules targeted against myostatin or its receptors [53]) is approaching the point of clinical use [54]. In the adipose tissue, myostatin inhibition [55] leads to an increase in brown adipose tissue characteristics. As novel weight loss compounds in the form of incretin receptor agonists (RA) (such as semaglutide or tirzepatide) have been associated with skeletal muscle loss, the beneficial effect of myostatin inhibitors on skeletal muscle mass and adipose tissue has led to the conception of animal trials utilizing the combination of myostatin inhibitors and incretin RA, which are already showing promising results [56].

In spite of their therapeutic potential, more safety studies are necessary, however, as myostatin seems to play an important role in connective tissue homeostasis. Experiments in myostatin-deficient mice have shown worse structural properties and lower fibroblast density in connective tissue, both of which have been associated with higher maximum stress, lower maximum strain, and increased stiffness [57]. Furthermore, a higher concentration of myostatin was found at the site of regenerating myotendinous junctions in injured mice [58], while the application of myostatin during wound healing led to accelerated proliferation and growth of early tissue, although it had no effect on later connective tissue repair processes [59]. The same study has also shown that myostatin is more significantly expressed in the intact loaded tendon [59]. However, some studies point towards a more neutral [57] or negative effect of myostatin on connective tissue, as myostatin inhibitors have been reported to positively affect bone density and architecture in mice when combined with physical activity [60]. This is corrobo-rated by a higher reported myostatin concentration at the site of bone injury in murine models, with myostatin application lowering the chondroblast density [61]. Taken together, these studies paint a conflicting picture of the role of myostatin in injury healing, as myostatin seems to have a positive effect on tendon regeneration [57], in contradiction to its negative role in bone regeneration [61].

## Activin A

Activin A is a member of the TGF-β family with a wide variety of functions in the human body, as it is involved in the development of male and female gonads, nervous system, skeletal system, immune system, nephrogenesis, etc. [62]. Due to its various effects in embryogenesis, the specific effect of activin A is significantly influenced by the tissue niche, making the description of its activity particularly difficult [63].

As a myokine, activin A has a high degree of similarity to myostatin in multiple respects, particularly in its mechanism of action, which is mediated by the same receptors (ALK4 and ALK5), and contributes to the creation of a negative feedback loop in skeletal muscle [64].

Activin A is expressed in skeletal muscle in resting conditions, with a significant increase after muscle injury in mice, suggesting an unknown role of activin A in the response of skeletal muscles to injury and wound healing [64]. Although its specific role in muscle regeneration has not yet been fully described, inhibition of activin A in mice leads to increased regeneration capacity *in vitro* [64]. Even in an uninjured murine muscle, activin A leads to progressive muscular atrophy *in vivo* through multiple pathways, including the p38β MAPK pathway [65]. It can therefore be postulated that inhibition of activin A can have a significant therapeutic benefit in sarcopenia, with a yet-unknown effect on muscular regeneration.

## Follistatin

As with all members of the TGF-β family, the effect of activin A and myostatin is directly related to the concentration of follistatin and inhibins, which exert an inhibitory effect on both groups. Although inhibins are not believed to play a direct role in the physiology of skeletal muscle, follistatin has been repeatedly shown to have a positive effect on skeletal muscle [66].

Although significantly elevated plasma levels of follistatin have been found after physical exercise, available data point to a hepatic origin in response to muscle contraction through an unknown intermediary, as no increase in follistatin was found in blood taken from the exercised leg or in murine skeletal muscle samples [67]. Published trials have shown only a modest decrease in circulating levels of follistatin in mice with impaired production of muscle-derived FST [68]. Thus, in the absence of any contradictory data, it is likely that the release of muscle-derived follistatin primarily has a local paracrine effect.

Although the primary mechanism of action of follistatins is believed to be inhibition of myostatin, experiments have shown that follistatin is capable of exerting its action even in myostatin-null mice [69].

Follistatin has been shown to have a significant positive effect on skeletal muscle mass, as injection of AAV-mediated follistatin has been associated with a significant increase in muscle volume not only in the injected skeletal muscle, but also in remote locations [70]. Lower follistatin production has also been associated with lower muscle fiber diameters and slower regeneration rate, highlighting its role in maintaining adequate muscle quality [66].

In conclusion, while follistatin has been shown to play a role in skeletal muscle metabolism and contributes to muscle hypertrophy, the extent of muscle-muscle or muscle-connective tissue crosstalk cannot be estimated at the moment due to the significant production of this exerkine by other organs in response to exercise [67].

## Musclin/osteocrin

A myokine with a high degree of similarity to natriuretic peptides, capable of binding to natriuretic peptide clearance receptors, musclin (also known as osteocrin) in skeletal muscle is associated with an increase in exercise capacity through improved oxidative capacity [71]. Uniquely among myokines, musclin seems to be capable of exercising its activity only in the presence of atrial natriuretic peptide (ANP). As ANP by itself has been associated with increased physical capacities and is capable of exerting its action even in the absence of musclin, musclin has been postulated to be a mediator of peripheral effects of ANP in response to physical training, with musclin deficiency leading to decreased physical endurance [71].

Although musclin is constantly expressed in skeletal muscle, physical activity leads to a significant increase in its plasmatic levels [71]. Although the available data do not show a direct effect on muscle hypertrophy, musclin has been associated with accelerated regeneration of skeletal muscle and has been shown to have a positive effect on senescent cells in murine skeletal muscle [72]. Musclin also has a function in adipose tissue, as it, *in vitro*, contributes to lipolysis and attenuated lipogenesis in murine adipose tissue through PKA/p38 signaling pathway [73].

However, the concept of the beneficial metabolic effect of musclin has been contradicted *in vivo*, as elevated plasma concentrations of musclin have been associated with obesity and metabolic disorders in both humans and rodents [74]. In mice, the primary site of musclin binding appears to be inguinal white adipose tissue, a region with a high concentration of beige adipose tissue. Interestingly, musclin concentration has been found to be temperature dependent, as it was found to be elevated in the skeletal muscle of mice exposed to 30°C [74]. The same study has also shown a negative effect of musclin on adipose tissue thermogenesis, raising questions about the role of musclin in exacerbating obesity through a reduced basal metabolism rate. As musclin has also been found to be associated with a lower grip strength and dynapenia in elderly adults, musclin *in vivo* appears to be a negative factor in metabolic health, in contradiction to its seeming positive effect *in vitro* [75].

## Leukemia inhibitory factor (LIF)

Although LIF was originally described to have an effect on myeloid cell lineage, it has also been shown to have a role in inducing the acute phase effect in hepatocytes, to inhibit lipoprotein lipase activity in adipose tissue, and to provide hypertrophic stimulus in skeletal muscle [76]. LIF is a member of the IL-6 superfamily with many functional similarities to IL-6, including the shared signal transduction through gp130 and the exercise-dependent origin in skeletal muscle [76]. However, LIF activity is mediated through a different receptor complex (trimer of the LIF, alpha, and gp130 receptor), and although physical activity leads to a local increase in LIF concentration in skeletal muscle, it has not been shown to be released into the systemic circulation [77].

LIF has been shown to be involved in muscle hypertrophy, as the addition of murine LIF to myotubes *in vitro* leads to an accelerated proliferation of satellite cells. [78] The important role of LIF in skeletal muscle hypertrophy has also been demonstrated *in vivo*, since LIF knockout in the murine model leads to a significantly attenuated response of the skeletal muscle to load, which can be rescued by LIF application [79]. Interestingly, skeletal muscle injury in mice leads to a significant local increase in LIF concentration, which implies an important role in skeletal muscle healing, as LIF infusion has also been found to accelerate skeletal muscle regeneration [80]. LIF has also been shown to have therapeutic potential, as the genetic modification of leukocyte progenitors by a LIF transgene has been found to have a therapeutic benefit in a murine dystrophic muscle model [81].

LIF also appears to have an important effect on adipose tissue metabolism and fatty acid distribution, as peripheral administration of LIF in mice led to a decrease in adipose tissue mass and an increase in adipocyte lipolysis [82], which was correlated with increased proliferation of adipose tissue and a decrease in liver triglyceride content in a murine model of LIF receptor alpha knockout [83]. However, current data do not point to a direct effect of muscle-derived LIF on adipose tissue.

## Fibroblast growth factor 21 (FGF-21)

FGF-21 is a cytokine expressed in multiple organs and tissue types, including liver, white adipose tissue, heart, brown adipose tissue, and skeletal muscle, exerting its action through FGF receptors [84]. While the FGF-21 expression in skeletal muscle is low under resting conditions, pathological conditions have been found to significantly increase the amount of FGF-21 both locally and systemically [84].

The role of FGF-21 in skeletal muscle appears to be as the primary mediator of the stress response, as although murine FGF-21 knockout has not been associated with significant adverse effects on skeletal muscle *in vivo*, the same knockout also led to attenuation of muscular atrophy in response to fasting conditions. Furthermore, FGF-21 overexpression has been found to decrease muscle mass and increase muscular mitophagy [84], implicating a significant role of FGF-21 in the induction of the starvation response in skeletal muscle through skeletal muscle atrophy and amino acid release [85]. Interestingly, FGF-21 has also been found to participate in conversion of fast-twitch fibers to more endurance-oriented slow-twitch fibers [86]. As fasting conditions in mice lead to more pronounced atrophy of fast-twitch fibers compared to their slow-twitch counterparts [87], FGF-21 may have a function in skeletal muscle adaptation to starvation, although more research is necessary to fully uncover its role.

Regarding metabolic health, acute elevation of FGF-21 leads to increased insulin sensitivity through its action in brown adipose tissue [88]. However, even though FGF-21 is released systemically during stress [84], it is currently unclear whether muscle-derived FGF-21 has a direct paracrine effect on adipose tissue.

## Endocrine action of myokines in the body outside of skeletal muscle

**Fig. 3:**
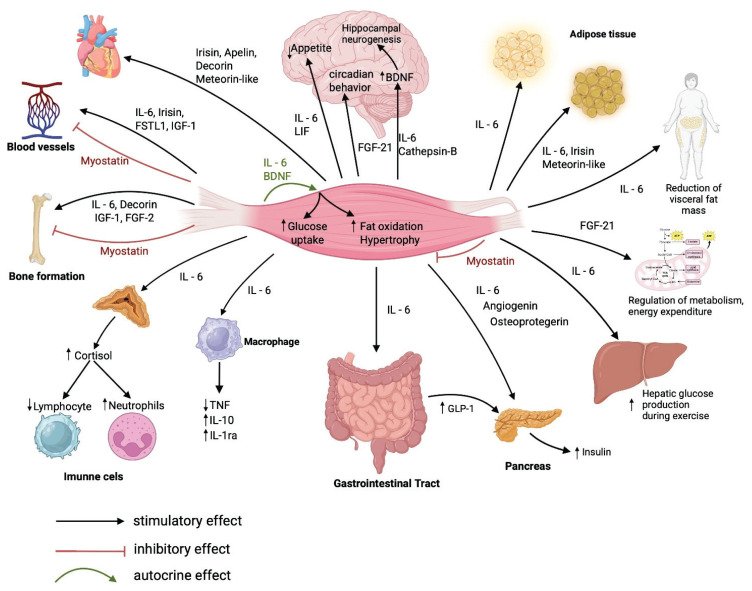
Muscle as an endocrine organ. BDNF – brain-derived neurotrophic factor, Cathepsin-B – cathepsin B, FGF-2 – fibroblast growth factor 2, FGF-21 – fibroblast growth factor 21, FSTL1 – follistatin-like 1, GLP-1 – glucagon-like peptide-1, IGF-1 – insulin-like growth factor 1, IGF-2 – insulin-like growth factor 2, IL-1ra – interleukin-1 receptor antagonist, IL-6 – interleukin-6, IL-10 – interleukin-10, LIF – leukemia inhibitory factor, TNF – tumor necrosis factor.

Despite significant advances, there are still many gaps in our understanding of a significant portion of myokines. While myokines are involved in autocrine regulatory mechanisms within muscle metabolism, their influence is not limited to skeletal muscle. As myokine receptors are widely expressed in many tissues and organs, including the heart, liver, adipose tissue, pancreas, bone, immune system, and nervous system, it seems likely that myokines play a highly variable role in corporal regulation [89]. A basic overview of the influence of known myokines on organ systems of the body is shown in Figure 3.

## Myokines in relation to organs of energy homeostasis and metabolism

Myokines are primarily produced and released from the skeletal muscle during physical exercise, which is associated with an increased need for metabolic resources. Available evidence implies a significant role of myokines in glucose homeostasis, partially exerted through their effect on the GI tract [90], liver [91] and the adipose tissue [92,93].

### Muscle-to-GI tract and pancreas crosstalk

IL-6 is one of the key myokines with a function in the GI tract, as IL-6 exerts a direct action on jejunal L cells, modulating the magnitude of the incretin effect on pancreatic β-cells by stimulating the release of GLP-1. IL-6 also has been shown to have a direct effect on pancreatic tissue *in vivo* by Ellingsgaard, as IL-6 leads to α-cell proliferation and a reduction of α-cell metabolic stress-induced apoptosis [90].

Additionally, recent data also point to a significant IL-6 role in modulating food intake, as IL-6 has been shown to have an effect on prolonging gastric evacuation by Pedersen’s group [94].

### Muscle-to-liver crosstalk

The liver is a paramount metabolic organ due to its role in nutrient processing and storing and is responsible for providing both energetic and structural substrates to other organs. In situations that increase the energy demand, such as muscular work, the liver provides additional glucose to cover the increased demand. Not surprisingly, some of the myokines are reciprocally involved in metabolic processes and in the regulation of energy homeostasis. The myokines that have been described as involved in hepatic metabolism so far include IL-6, Irisin, BAIBA (β-aminoisobutyric acid), Myonectin, and FGF21 [91].

### Muscle-to-fat-tissue crosstalk

Several myokines have been identified as important mediators of muscle-adipose tissue communication. Irisin drives the browning of white adipose tissue, particularly subcutaneous fat, by increasing UCP1 expression and thermogenesis [92,93]. In pioneering murine studies, it was demonstrated that even moderate increases in circulating irisin levels could transform energy-storing white adipocytes into “brown-like” adipocytes with increased energy expenditure. Diet-induced obese mice treated with irisin showed remarkable improvements in glucose tolerance and reduced insulin resistance, highlighting the therapeutic potential of irisin [92]. Major myokines influencing adipose tissue are shown in Table 2.

Novel research methods have expanded our understanding of the muscle secretome. A recent study developed a rapid centrifugation technique to isolate extracellular fluid from muscle and adipose tissues, identifying prosaposin (PSAP) as a previously unrecognized myokine and adipokine [95]. PSAP is up-regulated by PGC1α expression in muscles, increases in thermogenic fat during cold exposure, and stimulates thermogenic gene expression and mitochondrial respiration in primary fat cells [31].

The myokine repertoire affecting adipose tissue extends beyond these examples. IL-15 reduces the mass of adipose tissue [96], while meteorin-like (Metrnl) is triggered by resistance exercise and increases energy expenditure [93,97]. β-aminoisobutyric acid (BAIBA) induces browning of white adipose tissue [6,97] and myonectin regulates the uptake of fatty acids in response to changes in nutrients [96]. Myostatin functions primarily as a negative regulator of muscle growth [45]. Intriguingly, recent research using transgenic mouse models has revealed a new endocrine axis in which brown adipose tissue (BAT) can also secrete myostatin, affecting muscle function [45].

Physical activity profoundly modulates myokine production and secretion, establishing an anti-inflammatory environment that protects against chronic metabolic diseases [97]. One of the major metabolic benefits of exercise is the improvement of insulin sensitivity, which is particularly important in preventing insulin resistance and development of type 2 diabetes, especially in people with obesity or metabolic syndrome [11].

Beyond exercise, cold exposure represents another significant stimulus for myokine production and adipose tissue modification. In rodent studies, cold exposure induces shivering thermogenesis in muscle, which triggers the release of irisin and other myokines [98]. These cold-induced myokines promote browning of white adipose tissue and enhance non-shivering thermogenesis [99]. The combination of exercise and cold exposure may represent an optimal regimen to regulate myokine profiles and maximize metabolic benefits.

The crosstalk between skeletal muscle and adipose tissue, mediated by myokines, represents a critical mechanism to maintain metabolic homeostasis. Exercise-induced myokines transform adipose tissue metabolism, promoting a healthier phenotype characterized by increased energy expenditure and reduced inflammation. Understanding these complex interactions provides information on the mechanisms by which physical activity protects against metabolic disorders and may open new avenues for therapeutic interventions targeting myokine signaling pathways. The key regulatory conditions and their effects on the myokine profile and metabolic impact are presented in Table 3.

When critically evaluating the interpretation of myokine studies and experiments, it is, however, important to note that the research on myokines in the human body is complicated by their pleiotropic nature and multiple sites of origin outside of the skeletal musculature. As many of these molecules can be secreted into the circulation by different cell types both at rest and in response to physical exercise, it is often difficult to pinpoint the exact origin of the given molecule outside of more specialized and invasive experiments [5,18]. While some studies specify the presence and concentration of myokines at tissue level, a major proportion of the experiments rely on the plasma myokine levels, which reflect systemic levels [18]. Additionally, the histological and immunobiological analyses of the tissue samples do not accurately capture the dynamics of paracrine signaling, due to diffusion, fixation artifacts, and the loss of soluble factors during tissue processing [100].

## Conclusions

In the last two decades, significant discoveries have been made about the influence of physical activity and the production of large amounts of molecules in skeletal muscle. Movement activity and myokine release significantly reduce metabolic diseases. Physical activity has been shown to be important in the prevention of many chronic diseases, such as type 2 diabetes, cardiovascular disease, and cancer [11].

Identification of the muscle secretome provides new opportunities to understand the role of skeletal muscle in the regulation of metabolic function and how skeletal muscle is involved in the regulation of other organs. Myokines are involved in communication with most organs, such as adipose tissue, digestive tract, bone, and brain. In recent years, research on physical activity has provided new insights into how exercise affects the body at the molecular level. This knowledge has led to a re-evaluation of the role of skeletal muscle, which is now also seen as a secretory organ. Analysis of the muscle secretome has opened the way to a better understanding of the intercommunication between muscles and other body systems, while providing information on the mechanisms that keep muscles healthy.

Different myokines, signaling molecules secreted by muscles, affect not only muscles themselves but also distant organs such as adipose tissue, liver, pancreas, bone, and brain. For example, myostatin, LIF, IL-4, IL-6, and IL-7 play a role in muscle growth and the formation of new muscle fibers. Myokines such as BDNF and IL-6 contribute to fat burning, while myonectin and IL-6 affect lipid metabolism. BDNF is involved in the regeneration of muscle tissue after damage. IL-6 appears to be a multifunctional molecule – it interferes with the liver, adipose tissue, and the immune system, influences the interaction between the gut and pancreatic islets, and has anti-inflammatory effects. Other important substances include FSTL-1, which promotes vascular health, and the myokines irisin and FGF21, which stimulate the formation of brown adipose tissue.

According to recent findings, it seems that the main physiological role of the muscular secretome, and therefore myokines produced by skeletal muscle, is to improve functionality and protect muscle repair mechanisms during and after muscle exercise. They improve the functionality of myofibrils, fibroblasts, and immune cells in the working muscle, as well as the vasculature. The knowledge of myokines and other muscle-secreted molecules in the future will be crucial for the development of therapeutic approaches and drugs that can make significant inroads into the treatment of important metabolic diseases such as type 2 diabetes, cardiovascular disease, and age-related diseases such as sarcopenia.

## Figures and Tables

**Table 1:** Summary of reviewed myokines.

*Myokine*	Cytokine characterization	Target in skeletal muscle	Systemic release from skeletal muscle	Effect on skeletal muscle – cellular	Effect on skeletal muscle – functional	Other potential paracrine effects
*IL-6*	IL-6 superfamily	Membrane and soluble IL-6 receptor	Yes	Activation of satellite cells Increased glucose uptake Increased fatty acid oxidation	Acute high dose – muscle breakdown Long-term infusion – muscular atrophy Post-exercise levels – muscle breakdown	Free fatty-acid mobilization Reduction in visceral adipose tissue
*BDNF*	Neurotrophins	Tropomyosin receptor kinase B receptor	Controversial	Increased fatty acid-induced mitofission Reduced mitophagy Facilitation of neuromuscular connection Prevention of myosteatosis	Higher contraction force	Osteoblast activation Effect on adipokine secretion
*Irisin*	Fragment of FDNC5	Likely αVβ5	Yes	Proliferation of murine myoblasts Increased number of activated myoblast nuclei	Muscle regeneration Attenuation of muscular atrophy after denervation Increase in grip-strength	Adipose tissue browning Differentiation of adipocytes into beige adipose tissue Bone strengthening Proliferative and anti-inflammatory effect on tendons (Heat sensing in diabetic neuropathy)
*Myostatin*	TGF-β family	ALK 4, ALK5	Yes	Induces muscle protein breakdown through ubiquitin-proteasome system Increased autophagy Inhibits satellite cell activation Myotubular degeneration	Decrease in skeletal muscle mass	Conflicting role in the regeneration of muscular, skeletal, or tendon injury Adipose tissue regulation
*Activin A*	TGF-β family	ALK 4, ALK5	Unknown	Myofibrillar protein loss Muscular atrophy	Decrease in skeletal muscle mass	Conflicting role in regeneration of muscular, skeletal, or tendon injury
*Follistatin*	Glycoprotein	Myostatin and activin A	Unlikely	Antagonizing myostatin and activin A Skeletal muscle architecture maintenance Skeletal muscle regeneration	Increase in skeletal muscle mass	Possible positive effect on skeletal system
*Musclin (osteocrin)*	Natriuretic family	Natriuretic peptide clearance receptors	Yes	Skeletal muscle regeneration Senolytic effect on senescent cells	Increase in physical endurance	Possible negative regulator of thermogenesis Possible role in pathogenesis of obesity
*LIF*	IL-6 superfamily	LIF receptor alpha	No	Accelerated proliferation of the satellite cells Skeletal muscle regeneration	Increase in skeletal muscle mass	Decrease in adipose tissue mass Increase in adipocyte lipolysis
*FGF-21*	Fibroblast growth factor family	FGF receptors	Yes	Increase mitophagy Conversion of fast-twitch fibers to slow-twitch fibers	Skeletal muscle atrophy in fasting Decrease in skeletal muscle mass	Insulin sensitization of brown adipose tissue Adipose tissue beiging Induction of brown adipose tissue thermogenesis

ALK – activin receptor-like kinase; BDNF – brain-derived neurotrophic factor; FNDC5 – fibronectin-domain III containing 5; FGF – Fibroblast growth factor; IL-6 – interleukin-6; LIF – leukemia inhibitory factor.

**Table 2:** Major myokines influencing adipose tissue.

*Myokine*	Source/Regulation	Target	Primary Effects on Adipose Tissue
*Irisin*	Exercise, PGC1α-dependent, cleaved from FNDC5	White adipose tissue (esp. subcutaneous)	Drives browning of white adipose tissue Increases UCP1 expression and thermogenesis Improves glucose tolerance Reduces insulin resistance
*Musclin (Osteocrin)*	Skeletal muscle, elevated in obesity and thermoneutral conditions	Beige adipose tissue (*via* Tfr1 receptor)	Functions as negative regulator of thermogenesis Antagonizes thermogenic induction Worsens diet-induced obesity when overexpressed
*IL-6*	First identified myokine, increases upto 100-fold during exercise	Multiple tissues including adipose tissue	Creates anti-inflammatory environment Stimulates anti-inflammatory cytokines(IL-1ra, IL-10) Inhibits pro-inflammatory factors (TNF-α) Regulates cold-induced UCP1 expression
*IL-15*	Skeletal muscle	Adipose tissue	Reduces adipose tissue mass Promotes muscle protein metabolism
*Metrnl*	Triggered by resistance exercise and cold exposure	Adipose tissue	Increases whole-body energy expenditure Induces immune cytokines that promote thermogenic genes
*BAIBA*	Skeletal muscle during exercise	White adipose tissue	Induces browning of white adipose tissue Improves insulin resistance
*Myonectin*	Skeletal muscle in response to nutrient changes	Adipose tissue	Regulates fatty acid uptake
*Myostatin*	Primarily skeletal muscle, also brown adipose tissue (BAT)	Muscle and adipose tissue	Negative regulator of muscle growth Suppresses irisin, affecting VAT browning Downregulated by exercise and cold exposure
*PSAP*	Upregulated by PGC1α expression in muscle	Adipocytes	Stimulates thermogenic gene expression Enhances mitochondrial respiration
*FGF21*	Skeletal muscle *via* PI3-kinase/Akt1 pathway	Adipose tissue	Enhances lipolysis and thermogenesis Promotes VAT browning More related to non-shivering thermogenesis

Akt1 – serine-threonine protein kinase, BAIBA – β-aminoisobutyric acid, FDNC-5 – fibronectin-domain III containing 5, FGF21 – fibroblast growth factor 21, IL-1 – interleukin 1, IL-10 – interleukin 10, PGC1α – peroxisome proliferator-activated receptor-γ coactivator-1α, PI3 – phosphatidylinositol 3, PSAP – prosaposin, Tfr1 receptor – transferrin receptor 1, TNF – tumor necrosis factor, UCP1 – uncoupling protein 1, VAT – visceral adipose tissue.

**Table 3:** Key regulatory conditions.

*Condition*	Effect on Myokine Profile	Metabolic Impact
*Exercise*	↑ Irisin, IL-6, IL-15, Metrnl, BAIBA, FGF21 ↓ Myostatin	Promotes VAT browning Creates anti-inflammatory environment Improves insulin sensitivity
*Cold Exposure*	↑ Irisin, FGF21 ↓ Musclin, Myostatin	Enhances thermogenesis Increases energy expenditure
*Obesity*	↑ Musclin, pro-inflammatory factors ↓ Anti-inflammatory myokines	Reduced VAT browning Chronic inflammation Insulin resistance

BAIBA – β-aminoisobutyric acid, FGF21 – fibroblast growth factor 21, IL-6 – interleukin-6, IL-15 – interleukin-15, Metrnl – meteorin-like protein, VAT – visceral adipose tissue.
